# A Study on Solubilization of Poorly Soluble Drugs by Cyclodextrins and Micelles: Complexation and Binding Characteristics of Sulfamethoxazole and Trimethoprim

**DOI:** 10.1100/2012/718791

**Published:** 2012-04-29

**Authors:** Sinem Göktürk, Elif Çalışkan, R. Yeşim Talman, Umran Var

**Affiliations:** General Chemistry Division, Department of Basic Pharmaceutical Sciences, Faculty of Pharmacy, Marmara University, Uskudar, 34668 Istanbul, Turkey

## Abstract

The present study is focused on the characterization of solubilization of poorly soluble drugs, that is, sulfamethoxazole (SMX) and trimethoprim (TMP) by cyclodextrins (*α*-, *β*-, and *γ*-CDs) and anionic surfactant sodium dodecyl sulfate (SDS). The phase solubility diagrams drawn from UV spectral measurements are of the A_L_ type and indicate an enhancement of SMX and TMP solubility in the presence of CDs. Complex formation tendency of TMP with CDs followed the order: *γ*-CD > *β*-CD > *α*-C. However, the complex formation constant values, for SMX-CD system yielded the different affinity and follow the order: *β*-CD > *γ*-CD > *α*-CD. With taking into consideration of solubilization capacity of SDS micelles, it has been found that the solubility enhancement of TMP is much higher than that of SMX in the presence of SDS micelles. The binding constants of SMX and TMP obtained from the Benesi-Hildebrand equation are also confirmed by the estimated surface properties of SDS, employing the surface tension measurements. In order to elucidate the solubilization characteristics the surface tension measurements were also performed for nonionic surfactant Triton X-100. Polarity of the microenvironment and probable location of SMX and TMP were also discussed in the presence of various organic solvents.

## 1. Introduction

**Figure 1:**
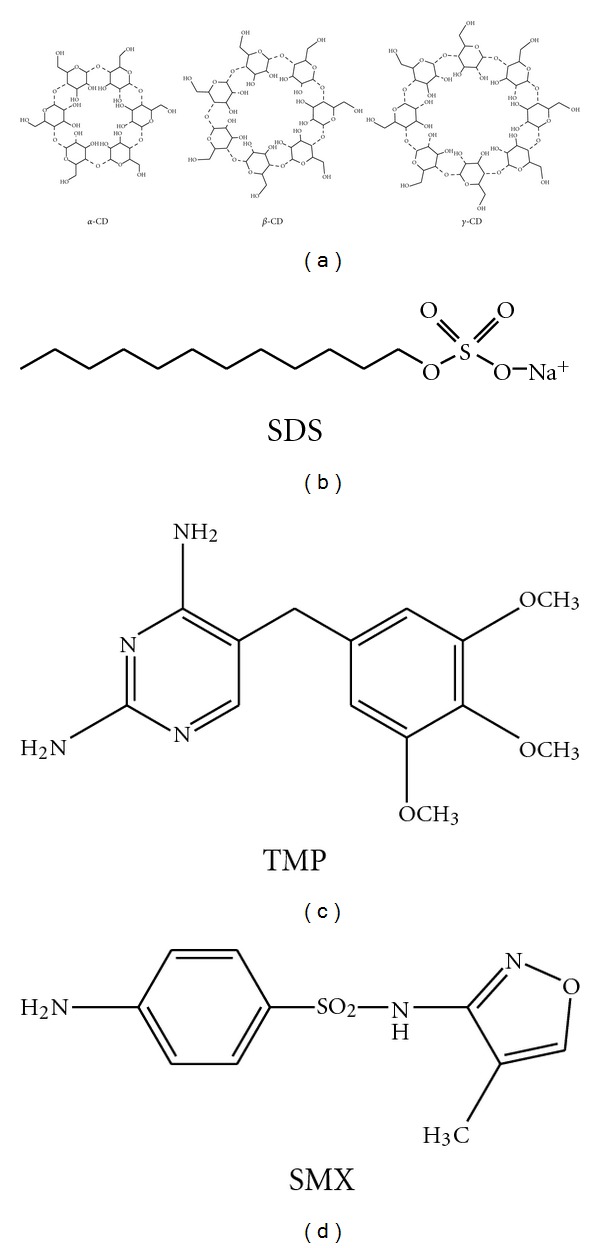
Chemical structures of *α*-CD, *β*-CD, *γ*- CD, SDS, TMP, and SMX.

Solubilization of poorly soluble drugs is one of the most important physicochemical properties for drug development since more than one-third of drugs are poorly water soluble or water insoluble. In order to prepare a liquid dosage formulation of these drugs, a solubilization technique is usually applied. The most commonly used techniques are pH adjustment, cosolvency, micellization, and complexation [1–4]. Cyclodextrins (CDs) are widely used as complexing agents for lipophilic and amphiphilic substances and functional excipients that have gained widespread use and attention because of their ability to solubilize, and in some instances stabilize, poorly water-soluble drug candidates enabling both oral and parenteral formulation [5–9]. CD molecules are peculiar cyclic oligomers of *α*-D-glucose; their shapes resemble truncated cones with primary and secondary hydroxyl groups located around their narrower and wider rim. The mostly used native cyclodextrins consist of 6, 7, and 8 glucose units, and, according to the number of monomers in the macrocycle, they are named as *α*, *β*, and *γ*-cyclodextrin, respectively. These molecules have a hydrophobic inner cavity, while the large number of hydroxyl groups on the outer surface makes them water soluble. Due to this special molecular structure, cyclodextrins are capable of forming inclusion complexes with many drugs and other compounds by taking up a lipophilic guest molecule (or its hydrophobic part) of the appropriate size into the cavity [10–12]. The chemical structures of CDs are shown in Figure 1. The ability of the CDs to form inclusion complexes with a variety of organic compounds is based on their ability to provide a hydrophobic cavity in aqueous solution for a hydrophobic guest molecule or hydrophobic moieties in the guest molecule. Moreover CDs have been used as a model for proteins and enzymes. The pharmaceutical interest in CDs extends to enhance the solubility, chemical stability, and bioavailability of poorly soluble drugs to reduce the toxicity and control the rate of release of some drugs [13]. Complexation of water-insoluble or poorly soluble drugs with native or modified cyclodextrins has been studied by a large number of workers [14–17], and some of the reports generated on this topic have been reviewed [18–21].

Micellar solubilization is also a powerful alternative for dissolving hydrophobic drugs in aqueous environments. Because of its amphiphilic nature, micelles are known to play a vital role in many processes of interest in both fundamental and applied sciences. Micellar system can solubilize poorly soluble drugs increasing their bioavailability, and they can be used as a model system for biomembrane, as well as drug carriers in numerous drug delivery and drug targeting systems. Micelles are known to have an anisotropic water distribution within their structure. In other words, the water concentration decreases from the surface towards the core of the micelle, with a completely hydrophobic (water-excluded) core. Consequently, the spatial position of a solubilized drug in a micelle will depend on its polarity: nonpolar molecules will be solubilized in the micellar core, and substances with intermediate polarity will be distributed along the surfactant molecules in certain intermediate positions. Surfactants such as sodium dodecyl sulfate (SDS) are used as excipients and usually added to the formulation to facilitate the preparation, patient acceptability, and functioning of the dosage form [4, 22]. The SDS molecule contains a 12-carbon-saturated alkyl chain bound to a negatively charged sulfonate head (OSO_3_^−^), and therefore, it is structurally comparable to many biosurfactants for which it is also used as a model. In aqueous medium, SDS (Figure 1) forms micelles which mimic biological membrane system as a model of globular protein and capable of solubilizing hydrophobic molecules resulting in a high local concentration. From this point of view, the interactions of drug with surfactants provide an insight into more complex biological processes such as the passage of drugs through cell membranes. The fundamental event in the interaction of drugs with biological tissues at the molecular level is their binding to membranes. A number of studies have been dedicated to drug/surfactant interactions [23–27]. This is an important issue because it relates to the mechanism of drug action. Therefore, the study of surfactant micelles and their role in pharmacy is of paramount importance, especially with respect to their ability of solubilizing hydrophobic drugs [15, 28–32]. Despite the large number of articles devoted to the interaction of drugs by surfactants or cyclodextrins, the studies that combine spectroscopy with surface tension measurements under identical experimental conditions are still missing. The aim of this work is to improve the effects of different types of excipients such as surfactant and cyclodextrins used in pharmaceutical applications on the solubilization and interactions of poorly soluble drugs in aqueous media. To more precisely analyze and evaluate interactions of drugs in different media, surface tension data are compared for the SDS and CDs in the presence of SMX and TMP in aqueous media. The dependence of SMX and TMP absorption spectra on the SDS and CDs concentration leads to interesting correlations with the surface tension measurements in terms of type of solubilization. For example, the solubilization of drug may not only involve incorporation into the micellar interior or cyclodextrins cavity but may also be substantially due to adsorption at the micelle-water interface or outer surface of cyclodextrins. Therefore, in this work, poorly soluble drugs sulfamethoxazole (SMX) and trimethoprim (TMP) were chosen as model to study their solubilization characteristics in the presence of SDS micelles and CDs (*α*, *β*, and *γ*-CD) in aqueous media at 298 K. The chemical structures of SMX and TMP are shown in Figure 1. SMX and TMP possess poor solubility in water; that is, S_SMX_= 600 mg L^−1^(≈2.368 × 10^−3^ M) and S_TMP_ = 400 mg L^−1^ (≈1.377 × 10^−3^ M). The combination antibacterial product containing SMX and TMP in a fixed 5 : 1 ratio is well known as Co-trimoxazole. Keeping in mind the scope of the study on solubilization of poorly soluble drugs SMX and TMP by SDS micelles and *α*-, *β*-, and *γ*-CDs, the present study is divided into four sections. In the first section, the interaction of fixed concentration of SMX and TMP (4.0 × 10^−5^ M) with SDS and CDs is discussed using the Benesi-Hildebrand equation to determine the binding constants of SMX and TMP in different environments. The concentration of 4.0 × 10^−5^ M was chosen as a common concentration to study interaction of two drugs with SDS and CDs to compare their binding tendency. At this concentration both SMX and TMP are freely soluble in water. The changes in surface tension of SDS and CDs in the presence of SMX and TMP are addressed in the second section to determine the surface excess of SDS by using the Gibbs adsorption equation. In the third section, the performance and effect of SDS micelles and CDs on the solubilization of SMX and TMP at their water insoluble concentrations range are compared and discussed using phase solubility diagrams. Finally, in order to gain further insight about the probable localization of poorly soluble drugs into the SDS and CDs, it has been monitored the variation of SMX and TMP absorption spectra in the presence of various organic solvents. Effect of medium polarity on the solubilization mechanism is also discussed in the present study.

## 2. Material and Methods

### 2.1. Materials

All the experiments were performed with analytical-grade chemicals and solvents. Trimethoprim [2, 4-diamino-5-(3', 4', 5'-trimethoxybenzyl pyrimidine)] (C_14_H_18_N_4_O_3_) and sulfomethoxazole [4-amino-N-(5-methylisoxazol-3-yl)-benzenesulfonamide] (C_10_H_11_N_3_O_3_S) were purchased from Sigma (Germany), respectively. *α*-, *β*-, and *γ*-cyclodextrins were obtained from Sigma-Aldrich. Sodium dodecyl sulfate and Triton X-100 were Sigma (Germany) products. Doubly distilled conductivity water was used for solution preparation.

### 2.2. Instruments

The spectroscopic measurements were performed on a double-beam UV-Vis spectrophotometer (Shimadzu UV-2100 S) equipped with a matched pair of cuvettes of 10.0 mm path length in a water-jacketed thermostatic cell holder. The reproducibility for *λ*_max_ of spectra was ±0.1 nm. All measurements were done at least in triplicate. The surface tension measurements were performed with KSV Instruments computer-controlled tensiometer, model Sigma 701 (Helsinki, Finland) employing the Wilhelmy plate method. The plate was cleaned by washing with doubly distilled water followed by heating in an alcohol flame. All surface tension values reported here are mean quantities of at least three measurements performed at 298 K. The standard deviation of the mean never deviated ±1.2% of the mean. The precision of the surface tension apparatus was 0.1 mN/m.

### 2.3. Methods

The interactions of SMX and TMP with SDS and CDs as well as solubilization experiments have been performed by a combination of surface tension measurements and UV-absorption spectrophotometry.

#### 2.3.1. Spectrophotometric Measurements

Changes in absorbance of SMX at 265 nm and TMP at 274 nm were observed and determined linear between the concentrations of 1.0 × 10^−5^–1.0 × 10^−4^ M (*R* ^2^ = 0.9989). Absorption spectra of SMX and TMP in aqueous solution with a varying wide concentration range of SDS, *α*-, *β*-, and *γ*-CDs have been recorded at 298 K (±0.1), keeping the concentration of drugs (4.0 × 10^−5^ M) fixed in each one. Binding constants were determined from the absorbances of a series of solutions containing a fixed concentration of drugs and increasing concentration of SDS, *α*-, *β*-, and *γ*-CDs.

#### 2.3.2. Surface Tension Measurements

Surface tension of aqueous solutions of CDs and surfactant in the absence and presence of fixed concentrations of SMX and TMP were measured at 298 K by computer-controlled Wilhelmy plate method in order to determine maximum surface excess concentration of surfactants and CDs in the presence of each drug.

#### 2.3.3. Solubility Measurements

Phase-solubility studies of SMX and TMP in aqueous solutions of CDs and SDS were carried out according to the Higuchi-Connors procedure [33]. Various amounts of SDS and CDs were generally dissolved in distilled water, and excess amounts of SMX and TMP were loaded in glass vials. The vials were shaken in a temperature-controlled room at 298 K for 24 h to achieve the equilibrium. Appropriate aliquots were then withdrawn and filtered appropriately diluted with distilled water, and the total concentration of the drugs in the filtrate was analyzed by UV absorbance spectrum. The absorbances at 274 nm for TMP and 265 nm for SMX were measured, in order to determine the concentration of the dissolved drugs.

### 2.4. Theoretical Background

#### 2.4.1. Determination of Binding Constants of Drugs with CDs and SDS Micelles

The binding constants of drugs to micelle and/or cyclodextrins can be quantitatively determined using the Benesi-Hildebrand equation [34] in the following modified form [25, 35, 36]:

(1) $$\frac{\left[\text{D}\right]l}{\text{A}-{\text{A}}_{0}}=\frac{1}{\Delta \varepsilon }+\frac{1}{K_{b}\left[\text{C}\right]\left(\Delta \varepsilon \right)\;},$$

where [D] and [C] represent the concentrations of drug and CD or SDS micelles, respectively. In the case of SDS micelles, C is micellized surfactant concentration; that is, [C] = (total surfactant concentration-CMC). *l* is the optical path length of the solution. A and A_0_ are the absorbances of drug in the absence and presence of SDS or CDs, respectively. Δ*ε* is the difference in molar absorption coefficients between complexed/bound and free drug. The plot of [D] l/(ΔA) against 1/[C] was found to be linear in all cases.

#### 2.4.2. Determination of Surface Excess Amount and Minimum Area per Molecule

On the basis of a plot of the surface tension, *γ*, as a function of the equilibrium concentrations of SDS and CDs in the presence of fixed concentrations of SMX and TMP, the changes of surface properties, namely, maximum surface excess concentration (Γ_max_) and minimum area per molecule (A_min_), can be determined by application of the Gibbs adsorption isotherm [4]:

(2) $$\Gamma_{\text{ max}}\;=-\frac{1}{\text{RT}}\left(\frac{d\gamma }{d\;\ln\text{C}}\right),$$

where R is the gas constant, T is the temperature in Kelvin, and C is the concentration of SDS or CD. Changes in minimum surface area per molecule can be obtained from the maximum surface excess concentration at the air-solution interface, A (Å^2^ molecule^−1^), and were evaluated from

(3) $$\text{A}=\frac{1}{{\text{N}}_{\text{ A}}\Gamma_{\text{ max}}},$$

where N_A_ is the Avogadro constant.

#### 2.4.3. Drug Solubilization Using Cyclodextrins

Phase solubility diagrams were analyzed to obtain estimates of the complex formation constants of soluble complexes described earlier [33, 37]. The linear part of the experimental solubility isotherms suggests that in these equilibria CDs-SMX or CDs-TMP associates of 1 : 1 molar ratio are dominantly formed. The K**_1:1_** stability constants for associates of 1 : 1 molar ratio have been calculated using the approach offered by Iga et al. [38]:

(4) $$K_{1:1}=\frac{\text{slope}}{\left[S_{0}\left(1-\text{slope}\right)\right]},\;$$

where S_0_ is the solubility of the drugs at 298 K in the absence of cyclodextrins and slope means the corresponding slope of phase-solubility diagrams, that is, the slope of the drug molar concentration versus CDs molar concentration graph.

#### 2.4.4. Drug Solubilization Using Surfactant

The solubility of a drug in the presence of a surfactant alone can be expressed by considering the two-phase model wherein it is assumed that the micellization is seen only above the critical micellar concentration (CMC) and that the monomer concentration is constant irrespective of the total surfactant concentration above the CMC. For such a system, the total solubility of the drug in the presence of the surfactant (C_S_) is given by the following equation [31, 39]:

(5) $$S_{T}=K_{M}\left(C_{S}-C_{CMC}\right)+S_{0}.\;$$

Here, K_M_, C_S_, and C_CMC_ are the solubilizing capacity of micelles, surfactant concentration, and critical micelle concentration, respectively. Solubilizing capacity of micelles (K_M_) determined from the slope of the solubilization curve will be expressed by M^−1^ in this paper.

## 3. Results and Discussion

### 3.1. Interactions of SMX and TMP with SDS, *α*-, *β*-, and *γ*-CDs

**Figure 2:**
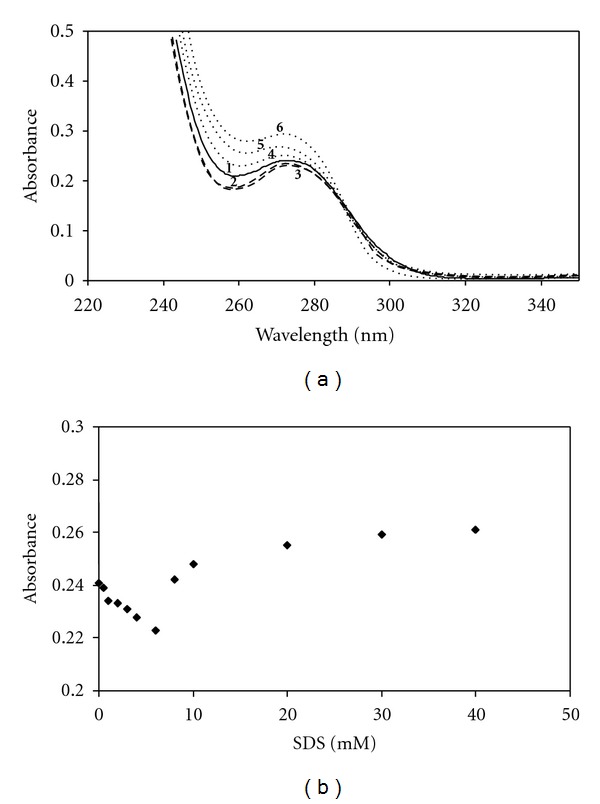
(a) Visible absorption spectra of TMP (4.0 × 10^−5^ M) at various concentrations of SDS at 298 K: (1) no SDS, (2) 2 mM, (3) 4 mM, (4) 8 mM, (5) 10 mM, (6) 20 mM SDS. (b) The absorbance change of 4.0 × 10^−5^ M TMP (below and above the CMC) with the concentration of SDS.

**Figure 3:**
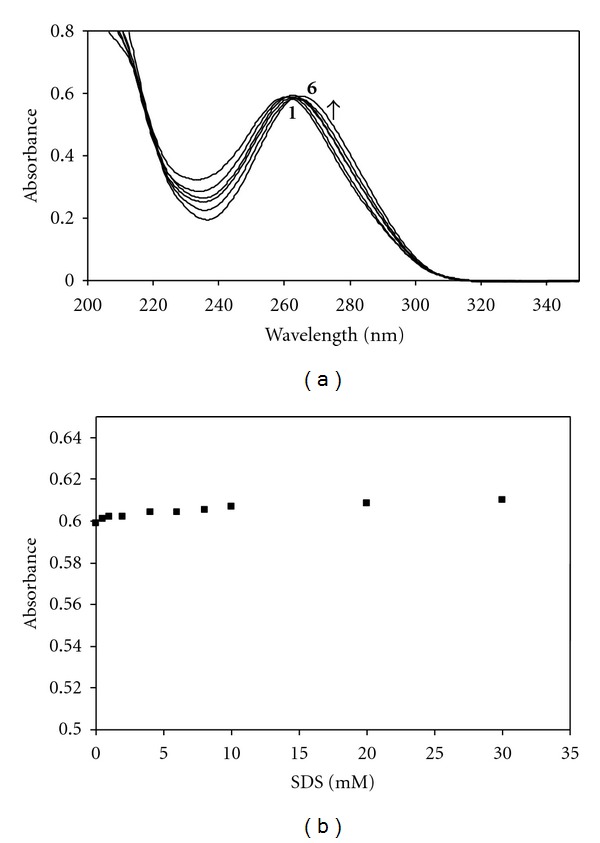
(a) Visible absorption spectra of SMX (4.0 × 10^−5^ M) at various concentrations of SDS at 298 K: (1) no SDS, (2) 2 mM, (3) 4 mM, (4) 8 mM, (5) 10 mM, (6) 20 mM SDS. (b) The absorbance change of 4.0 × 10^−5^ M SMX (below and above the CMC) with the concentration of SDS.

**Figure 4:**
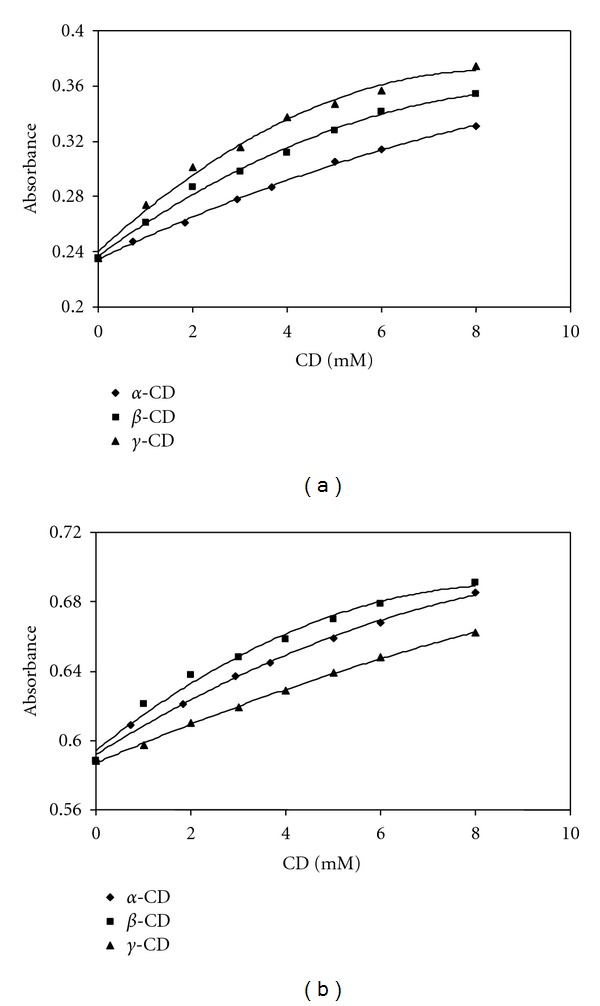
(a) The absorbance change of 4.0 × 10^−5^ M TMP with the concentrations of CDs. (b) The absorbance change of 4.0 × 10^−5^ M SMX with the concentrations of CDs.

**Figure 5:**
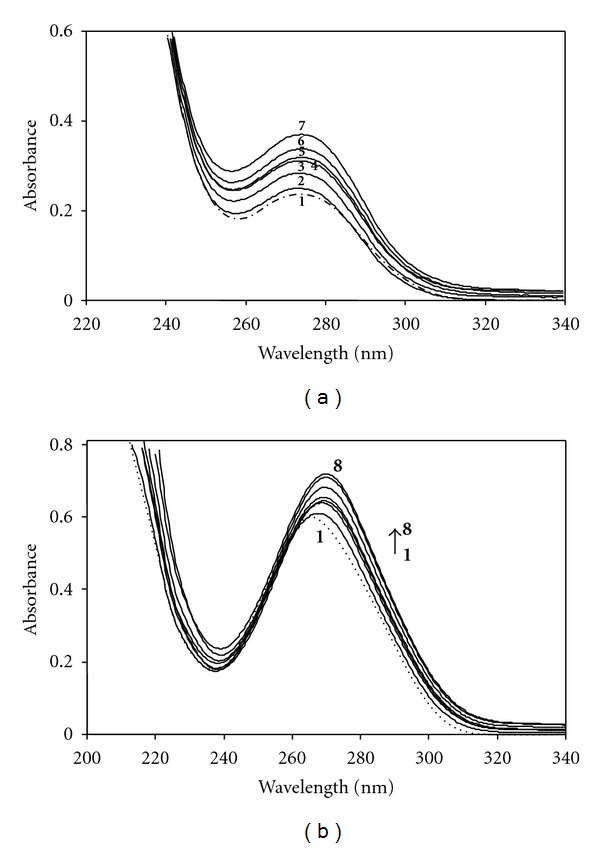
(a) Visible absorption spectra of TMP (4.0 × 10^−5^ M) at various concentrations of *γ*-CD at 298 K: (1) no *γ*-CD, (2) 1 mM, (3) 2 mM, (4) 4 mM, (5) 6 mM, (6) 8 mM, (7) 10 mM *γ*-CD. (b) Visible absorption spectra of SMX (4.0 × 10^−5^ M) at various concentrations of *β*-CD at 298 K: ↑ (1) no *γ*-CD, (2) 1 mM, (3) 2 mM, (4) 3 mM, (5) 4 mM, (6) 6 mM, (7) 8 mM, (8) 10 mM *γ*-CD.

**Figure 6:**
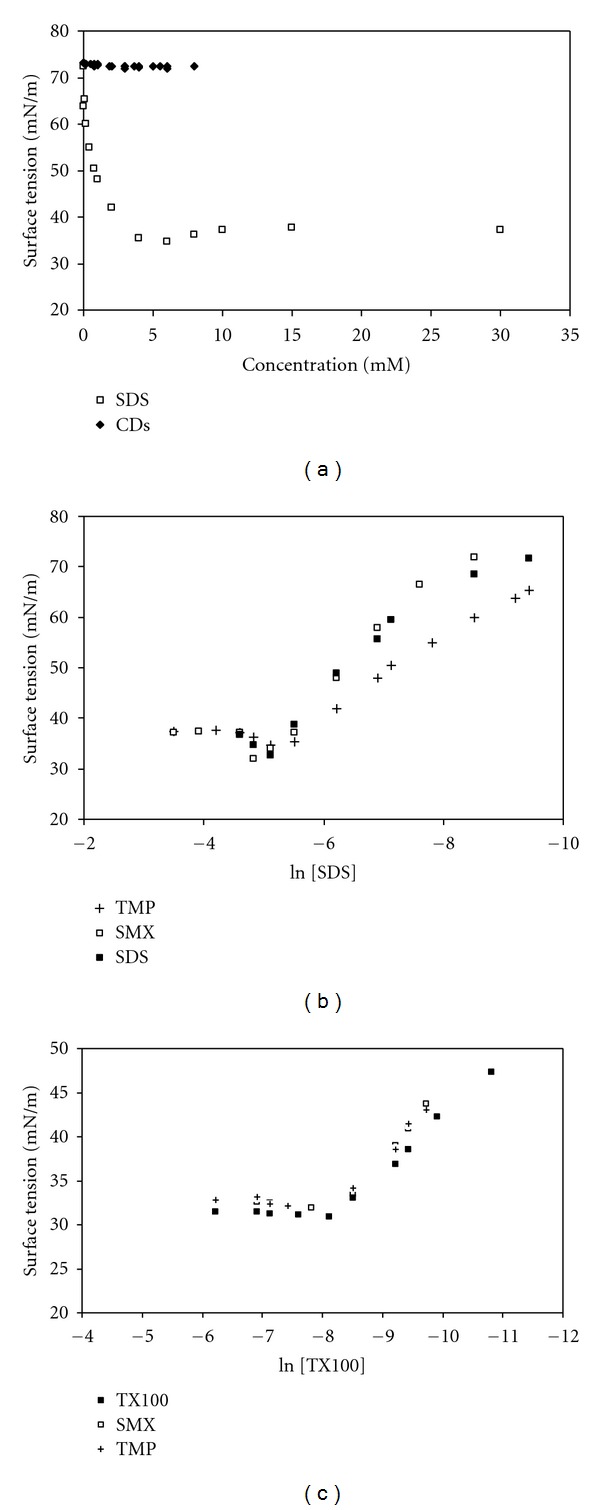
(a) Surface tension results for SDS and CDs in the presence of 4.0 × 10^−5^ M TMP at 298 K. (b) Surface tension versus ln (concentration) plots for SDS in the absence (▪) and presence of 4.0 × 10^−5^ M SMX (□) and TMP(+) at 298 K. (c) Surface tension versus ln (concentration) plots for TX100 in the absence (▪) and presence of 4.0 × 10^−5^ M SMX (□) and TMP(+) at 298 K.

SMX is an acidic compound, and the spectrum undergoes a hypsochromic shift with increasing pH, corresponding to the loss of a proton from the –SO_2_–NH– group, implying the existence of electron-withdrawing groups in the SMX anion [40, 41]. At pH values above 5.6, SMX exists predominantly as anionic species; at pH values between 1.7 and 5.6, SMX is uncharged, while at, pH values below 1.7, it is positively charged [42]. In our experimental conditions, the medium pH is above 5.7 where more stable SMX anions exist. TMP is a basic compound, and a proton is associated with the NH_2_ substituents in acidic solutions, but the spectrum shows a bathochromic shift as the pH increased [40]. The basic drug TMP and acidic drug SMX having the structures shown in Figure 1 exhibit the maximum absorption bands at 274 and 265 nm, respectively. The molar extinction coefficients (*ε*_0_) of TMP at 274 nm and SMX at 265 nm were calculated as 6.21 × 10^3^ and 15.17 × 10^3^ mol^−1^ dm^3^cm^−1^ in the concentration range of 1.0 × 10^−5^–1.0 × 10^−4^ mol dm^−3^ of the drugs at 298 K, respectively. The linear relation between absorbance and drug concentration (*R* ^2^ : 0.9998) indicates the validity of Beer's Law. The effect of anionic surfactant SDS, at the concentrations varied from 1.0 × 10^−5^ to 4.0 × 10^−2^ M (from premicellar to micellar region), on the absorption spectrum of SMX and TMP at fixed concentration of 4.0 × 10^−5^ M was studied. The change of absorption spectrum of TMP in the presence of various concentration of SDS was shown in Figure 2(a). TMP exists in cationic form in aqueous solution. As seen in Figure 2(a) the absorbance decreased with a small blue shift with the increase in SDS concentration up to 4 mM. It was observed that the increase in absorbance with the increase in SDS concentrations above the CMC, *λ*_max_ of TMP at 274 nm shifted to 270 nm. This observed blue shift and the increase in absorbance are a clear indication that TMP incorporate to SDS micelles.Figure 2(b) shows the relation between the absorbance of TMP and the various concentrations of SDS. A decrease in the absorbance observed at the concentrations below the CMC indicates the molecular complex formation between cationic drug TMP and anionic SDS molecules due to the electrostatic interactions. The increase in absorbance values with increasing surfactant concentrations above the CMC indicates association of TMP molecules with SDS micelles. As more drug molecules are incorporated to micelles, the absorbance values of *λ*_max_ reaches a limiting value and becomes almost constant; that is, the amount of solubilized TMP reaches saturation. The CMC value of SDS in the presence of TMP was determined spectrophotometrically and employed surface tension measurements as described previously [4, 36]. A good agreement was found between two methods, and the CMC of SDS was determined to be 4 mM in the presence of 4.0 × 10^−5^ M TMP. The absorption spectra of SMX in the presence of various concentrations of SDS are shown in Figure 3(a). At the concentrations below and above the CMC (8.0 mM), no spectral changes were observed, and the absorbance of SMX remained almost constant in the presence of SDS. As seen in Figure 3(b) there is a slight increase in absorbance, that is, only a very weak interaction occurred between SMX and SDS micelles due to the electrostatic repulsion. Under working conditions, SMX bears a net negative charge and SMX anion is dominant form. The spectral behaviour of 4.0 × 10^−5^ M TMP and SMX in aqueous solutions containing different concentration of CDs were studied. Figures 4(a) and 4(b) show the relation between the absorbance of TMP and SMX with the various concentrations of CDs, respectively. The increase in absorbance values with increasing CDs concentrations indicates association of drugs with CDs. Changes of absorption spectra observed for SMX and TMP in the presence of CDs were analogical, so in the present work only the figures are shown for the most effective CD on the complexation tendency of SMX and TMP (Figures 5(a) and 5(b)). A similar behavior, that is, a progressive enhancement in absorbance with the increase of CD concentration, was observed for all CDs.  Figure 5(a) shows UV spectra of TMP in the presence of various concentrations of *γ*-CD. No shift was observed in the *λ*_max_ of TMP at 274 nm when complexes are formed with *α*-, *β*-, and *γ*-CD. A similar behavior, that is, a progressive enhancement in absorbance of SMX, was also observed for SMX in the presence of various concentrations of *β*-CD and shown in Figure 5(b). From the spectral measurements, the binding constant values (K_b_) related to the extent of drug-SDS and/or drug-CDs interactions were calculated using the Benesi-Hildebrand equation and shown in Table 1. It has been found that the interaction between cationic TMP and anionic SDS micelles is the strongest because of the opposite charge of the components as expected. The lack of interaction of SMX with SDS supports this expectation, and the binding constant of SMX in the case of SDS micelles could not be calculated. The K_b_ values of SMX and TMP presented in Table 1 for the interaction of three types of CDs indicated the different affinities which follow the order as *β*-CD > *γ*-CD > *α*-CD in the case of SMX and *γ*-CD > *β*-CD > *α*-CD in the case of TMP. Before discussing on the different binding tendency of drugs to CDs as well as SDS micelles, it is worth mentioning the data obtained from surface tension measurements. In order to get a better insight in the mechanism of interaction of SMX and TMP with SDS and CDs, a thorough study of the surface tension change of SDS and CDs was performed in the presence of 4.0 × 10^−5^  M SMX and TMP. In this study, once the interfacial behaviours of SDS and CDs were analyzed in the absence of the drugs. *α*-, *β*-, and *γ*-CD did not show any surface property: the measured surface tensions of their solutions varied in the range of 71.6–72.0 mN*/*m only. As already reported in the literature native *α*-, *β*-, and *γ*-CD were not surface active [19, 43]. Since the same behaviour was observed for the surface tension measurements at various concentrations of SDS and CDs in the presence of 4.0 × 10^−5^ M SMX and TMP, only the results for TMP are shown in Figure 6(a) as a representative figure. As seen in Figure 6(a), no variation of the surface tension was observed for the CDs studied even with the increase in concentration of CD; on the contrary, the acting of SDS is different. The influences of SMX and TMP on surface properties of SDS are shown in Figure 6(b) plotted by the surface tension (*γ*) versus logarithm of concentration of SDS in the absence and presence of 4.0 × 10^−5^  M TMP and SMX. Using the concentration dependence of surface tension (Figure 6(b)), and the Gibbs equation, the quantities of adsorbing SDS and the apparent area of adsorbed SDS were determined in the absence and presence of SMX and TMP. The slope of surface tension (*γ*) versus ln concentration is proportional to the surface excess (Γ_max_) as apparent from (2).  Table 2 summaries the corresponding Γ_max_ and A_min_ values. As seen in Figure 6(b) the surface tension values of SDS in the presence of TMP are lower than those in the absence of TMP. On the contrary, the surface tension values of SDS in the presence of SMX is little higher than those in the absence of SMX. The data in Table 2 show that Γ_max_ value of SDS increased and A_min_ decreased in the presence of SMX while Γ_max_ value of SDS decreased and A_min_ increased in the presence of TMP. These findings support that electrostatic forces play an important role on the interaction between the drugs and SDS. The presence of TMP decreases the repulsion among head groups of SDS, and more TMP molecules can be adsorbed at the interface which is also confirmed by the higher value of A_min_; that is, TMP increased the adsorption of SDS at the interface. The higher the Γ_max_ value and the lower the A_min_ value for SDS in the presence of SMX also explains that the electrostatic repulsion forces take place in the interaction of anionic SMX and anionic SDS molecules compared to a pure SDS solutions. In order to gain better insight into the phenomena taking place at the interface, the surface tension measurements were also performed for nonionic surfactant Tritonx-100 (TX-100) in the presence of SMX and TMP at 298 K. The variation of surface tension as a function of TX-100 concentration in the absence and presence of 4.0 × 10^−5^  M SMX and TMP is also illustrated in Figure 6(c), and the data obtained from surface tension measurements presented in Table 2 in order to compare with the behaviour of SMX and TMP in the presence of ionic and nonionic micellar environments. It is also clearly seen that the surface tension values of TX-100 did not vary significantly as in the case of SDS with the addition of SMX and TMP; that is, the smaller increase of surface tension values of TX-100 was observed. Γ_max_ value of TX-100 increased, and A_min_ decreased in the presence of TMP as seen in Table 2. However, both Γ_max_ and A_min_ values of TX-100 remained almost the same as in the presence of SMX. It can be said that electrostatic forces take place in binding of SMX and TMP onto anionic SDS micelles, whereas hydrophobic interaction plays the main role in binding of SMX and TMP to nonionic TX-100 micelles. There is a reasonable agreement with the value obtained from spectrophotometric measurements due to the lack of interaction between SDS and SMX; that is, electrostatic repulsion forces overcome hydrophobic interaction.

### 3.2. Micellar Solubilization of SMX and TMP by SDS and CDs

**Figure 7:**
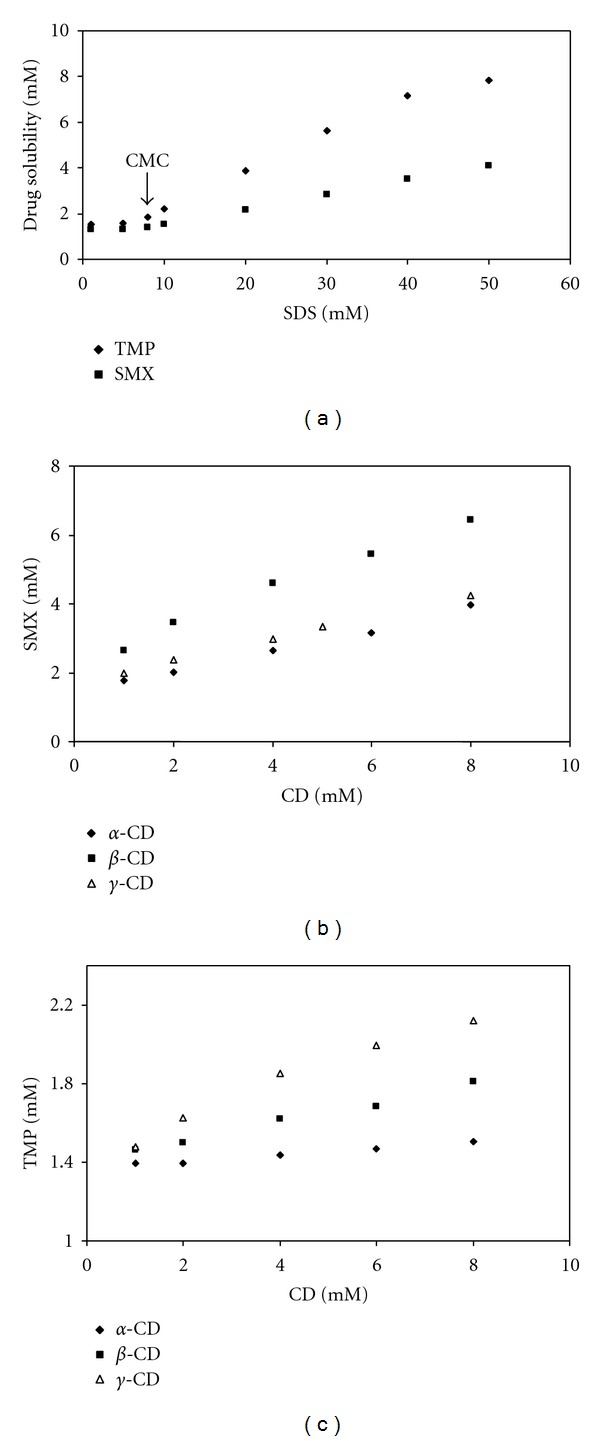
(a) Solubility curves of SMX and TMP as a function of SDS concentration at 298 K. (b) Phase solubility diagrams of SMX-CD systems at 298 K. (c) Phase solubility diagrams of TMP-CD systems at 298 K.

Figure 7(a) shows the solubility change of poorly soluble model drugs SMX and TMP as a function of SDS concentration. As seen in Figure 7(a), the plots of drug solubility against surfactant concentrations can be divided into two straight lines; one for below the CMC (8 mM) and the other above it. Above the CMC solubility of drugs increased with the increase in SDS concentration and good linearity was observed between the surfactant concentration and the solubility. The solubilization capacity K_M_ was determined to be 71.30 and 326.95 M^−1^ for SMX and TMP, respectively. These results obtained from (4) and given in Table 1 are compatible with the results of interaction studies discussed in the previous section. The solubility of SMX and TMP in the aqueous SDS solutions increased linearly with increase of the SDS concentration. Solubilization effect of SDS on TMP is stronger than that of SMX. This behaviour is a consequence of the electrostatic interactions between the negatively charged surfactant SDS and cationic drug TMP. As indicated in the interaction studies, the addition of TMP caused a decrease in the repulsive forces between the head groups of SDS molecules, contributing to the micellization process, and thus the CMC value of SDS decreased from 8 mM to 4 mM. This is also supported by the influence of SMX and TMP on the surface properties of SDS based on surface tension measurements.

### 3.3. Complexation of SMX and TMP by CDs

Figures 7(b) and 7(c) show the solubilization data of two model drugs SMX and TMP as a function of the CD concentrations, respectively. The solubility profile showed an increase in their solubility when the concentrations of CDs increase. The 1 : 1 drug/CD complexes are typical of association where a single drug molecule is in the cavity of a CD, with a stability constant (K_1:1_) for the equilibrium between the free and associated species. As seen in Figures 7(b) and 7(c), the solubility of SMX and TMP increased linearly as a function of CD concentration, and phase solubility studies revealed increase in solubility of the drug upon cyclodextrin addition, showing A_L_ type of graph with slope less than one indicating formation of 1 : 1 stochiometry inclusion complex. The corresponding stability (or complex formation) constants are listed in Table 1. The apparent solubilities (S_0_) of SMX (2.1 × 10^−3^ M) and TMP (1.4 × 10^−3^ M) in water obtained from phase solubility diagrams are close enough to the value of 2.40 × 10^−3^ M for SMX and 1.38 × 10^−3^ M for TMP as reported previously [42]. It is clearly seen that the stability of the inclusion complexes is in the different order for SMX and TMP. The stability constant K_1:1_ values for the TMP/CD system followed the order *γ*-CD > *β*-CD > *α*-CD. However, the different trend was observed in the case of SMX/CD system and followed the order *β*-CD > *γ*-CD > *α*-CD. Host complexation is primarily determined by the tightness of the fit, that is, by the size and shape matching between the guest and the CD cavity. CDs are designated *α*, *β*, or *γ* corresponding to 6, 7, or 8 glucopyranose units, with cavity diameters of 4.7–5.3, 6.0–6.5, and 7.5–8.3 Å, respectively [44]. Based on these dimensions *α*-CD can typically complex low-molecular-weight molecules or compounds with aliphatic side chains, *β*-CD will complex aromatics and heterocycles and *γ*-CD can accommodate larger molecules such as macrocycles and steroids. If the guest is the imperfect size, it will not fit properly into the cyclodextrins cavity and leads to a decrease in the host-guest interactions as well as the association constants [45]. The different ability of CDs to associate with TMP seems to be related with the fitting of the substrate into the CD cavity. The TMP molecule is approximately 7.39 Å in width and 11.972 Å in length [46]. Consequently, the internal diameters of the *α*-CD and *β*-CD cavities are too small for inclusion of the TMP molecules. The internal diameter of *γ*-CD cavity is suitable for accommodation of the TMP molecule. The relatively high K_1:1_ value for complexation of TMP with *γ*-CD indicates the existence of a better geometric fit than that with the *α*- or *β*-CD cavities. However, the different trend was observed in the case of SMX for the inclusion complex formation based on the dimensions of CDs. As data in Table 1 show, *α*-CD behaves similarly to *γ*-CD, but its ability to associate with SMX is relatively smaller. One of the most interesting results of the present study is the lack of complexation of SMX with *γ*-CD. However, the capability of *β*-CD to form complexes with SMX seems to be stronger than the inclusion complex formation tendency of SMX with *α*- and *γ*-CD. The reported molecular dimension of the SMX molecule is approximately 5.6 Å in width and 12.9 Å in length according to Comerton et al. [47]. The weak inclusion complex formation of SMX with *α*-CD can be explained with the smaller internal diameter cavity of *α*-CD. Thus, SMX molecule should be tightly packed into the *γ*-CD cavity. Although prediction of compound solubilization by CDs continues to be highly empirical, various historical observations permit several general statements. The formation of an inclusion complex with cyclodextrins is also classically caused by interactions such as hydrogen bonding with the OH groups at the periphery of the cavity, van der Waals interactions, and hydrophobic effects [48]. Hydrophobic interactions should play an important role for the stronger inclusion complex formation of SMX with *β*-CD as compared with that of *α*- and *γ*-CD. When we compare the aqueous solubility of *α*- (14.5%, w/v), *β*- (1.85%, w/v), and *γ*-CD (23.2%, w/v) in water [20], it can be resulted that *γ*-CD is the most soluble whereas *β*-CD is the least soluble in water; that is, *β*-CD is more hydrophobic than *γ*-CD. The interaction studies performed for the fixed concentration of SMX supports the behaviour of SMX in the presence of *β*-CD. The maximum absorption spectra of SMX shifted from 265 nm to 270 nm in the presence of *β*-CD while the maximum absorption spectra of SMX did not change significantly in the presence of *α*- and *γ*-CD.

### 3.4. Probable Location of TMP and SMX: Effect of Medium Polarity

**Figure 8:**
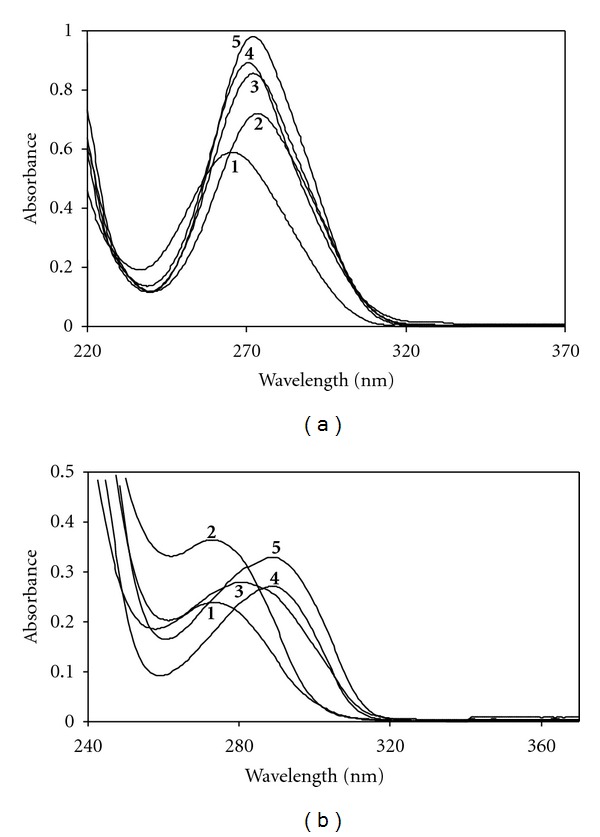
(a) Variation of visible absorption spectra of SMX (4.0 × 10^−5^ M) in the absence and presence of various solvents at 298 K: (1) in water, (2) Gly, (3) PG, (4) EOH, (5) EG. (b) Variation of visible absorption spectra of TMP (4.0 × 10^−5^ M) in the absence and presence of various solvents at 298 K: (1) in water, (2) Gly, (3) PG, (4) EOH, (5) EG.

As mentioned above the characteristics of the absorption spectra of SMX and TMP showed different trends in the presence of SDS, *α*-, *β*-, and *γ*-CD. These observations are indicative of change in the microenvironmental polarity of the drugs due to interactions with SDS and CDs. To gain insight into the localization of SMX and TMP in SDS and CDs, we have studied the spectrophotometric behavior of SMX and TMP in the presence of various organic solvents with different polarity such as ethanol (EOH), propylene glycol (PG), ethylene glycol (EG), and glycerin (Gly). Figures 8(a) and 8(b) present the absorption spectra of 4.0 × 10^−5^ M TMP and SMX, respectively, in the presence of various organic solvents for the sake of comparison also in water.

Comparison of different shifts in the presence of these organic solvents indicates the order of the polarity of the media follow the trend; water > Gly > PG > EG > EOH. This follows the same increasing order of *λ*_max_ of TMP. The *λ*_max_ of TMP at 274 nm in water shifted to 273, 281, 289, and 290 nm in the presence of Gly, PG, EG, and EOH, respectively. Our data showed that, upon going from the more hydrophilic to the more hydrophobic environment, TMP underwent a red shift in the absorption maximum (Figure 8(a)). The red shift is also a clear indication of the tendency of TMP to less polar environment. The *λ*_max_ values given in Table 1 support these findings in the presence of SDS and CDs. The parallelism between polarity of the medium and higher micellar binding constant demonstrates significantly that hydrophobic interactions play an important role in solubilization of TMP by SDS micelles beside electrostatic interactions. The small blue shift in the presence of Gly also supports the behaviour of TMP in the presence of SDS; that is, Gly is more hydrophilic than the other solvents studied. It can be said that TMP is located in the interfacial region of SDS micelles while in the case of CDs TMP prefers to locate in the hydrophobic region depending on the cavity size. As seen in Table 1, in contrast with TMP, the absorption maxima of SMX is red shifted by 5 nm in *β*-CD, whereas blue shifted 1–3 nm in *α*- and *γ*-CD compared with those in aqueous medium. These observed shifts show that SMX is transferred from a highly polar phase (H_2_O) to a less polar phase. This is supported with the red shift (from 265 to 272 nm) obtained when Gly, PG, EG, and EOH were used as solvent instead of water. As seen in Figure 8(b) no significant shifts of the *λ*_max_ of SMX were observed with the change of medium polarity. The higher binding constant of SMX determined in the presence of *β*-CD confirms the more binding tendency of SMX due to more hydrophobic character of *β*-CD. The inclusion complex formation of SMX with *β*-CD might be explained with only a part of the SMX molecule accommodated in the *β*-CD cavity and leaving the rest of the molecule protruding into water taking into consideration molecular geometry. The spectral behaviour of SMX in the presence of organic solvents indicates that SMX is willing to transfer more hydrophobic region. This is confirmed that the electrostatic repulsion forces are dominant on the interaction between SMX and SDS in aqueous media; that is, no significant shift was observed in the spectrum of SMX in the presence of SDS. The surface excess and molecular occupied area obtained from surface tension measurements also confirm these results (see S1 and S2 in Supplementary Material available online at doi:10.1100/2012/718791).

## 4. Conclusions

In this work the solubilizing ability of three different CDs (*α*-, *β*-, and *γ*-CD) and SDS were compared for two poorly soluble drugs, that is, SMX and TMP based on spectrophotometric and surface tension measurements. An extensive study on the solubilization characteristics of SMX and TMP in different environmental conditions has been presented at 298 K. To obtain more information about the nature and properties of the solubilization of the drugs by different structures for the fixed concentration of the drugs, as a model system for biomembranes, interactions of SMX and TMP with SDS and CDs were studied separately. The aqueous solubility of poorly soluble drugs can considerably be improved by micellar solubilization. The increases of the aqueous solubility of SMX and TMP in the presence of *α*-, *β*-, and *γ*-CD indicate that they are able to form inclusion complexes with different manner. Stability or equilibrium constants of the drug-CD complexes are important since this is an index of changes in physicochemical properties of a compound upon inclusion. Solubility of drugs in various media and the binding constants of drugs into CDs and SDS micelles were found to depend on drug characteristics as well. The solubilization of TMP with different CDs revealed that complex stability is affected by the geometric fit with CD. However, the higher tendency of SMX to complex with *β*-CD in aqueous solution is governed by the hydrophobic effect. Changes in surface properties of surfactants determined by surface tension measurements in the presence of drugs are in good agreement with the binding constants determined spectrophotometrically. Therefore, it is important to study the change in surface properties to understand the action mechanism of the drug especially in the case of surfactants. Another interesting point is that the different tendency was observed in the absorption maxima of SMX and TMP in the presence of various organic solvents. The *λ*_max_ values of SMX and TMP in the presence of CDs or SDS and organic solvents are in conformity with the localization of SMX and TMP for complexation and solubilization manner. As a conclusion, the presented results should be of special interest in the pharmaceutical researches in drug delivery systems.

## Supplementary Material

Inclusion complex formation of TMP/ g–CD and SMX/ b–CD *(*Figure 1) and micellar solubilization of TMP and SMX by SDS micelles *(*Figure 2).

## Figures and Tables

**Table 1:** K_1:1_ and K_M_ values for SMX and TMP in the presence of SDS and CDs at 298 K. K_b_ and *λ*_max_ (nm) values for fixed concentration of SMX and TMP (4.0 × 10^−5^ M) obtained in SDS and CDs.

	SMX				TMP			
	*K*_*b*_	*K*_1:1_	*K*_*M*_	*λ*_ max_/nm	*K*_*b*_	*K*_1:1_	*K*_*M*_	*λ*_ max_/nm
SDS	—	—	71.30	260	327.0	—	326.95	271
*α*-CD	30.15	279.21	—	264	54.09	13.02	—	273
*β*-CD	240.10	489.26	—	270	110.0	36.85	—	274
*γ*-CD	95.44	317.53	—	263	226.40	79.50	—	274

**Table 2:** Estimated surface excess (Γ_max_) and molecular occupied area (A_min_) for SDS and TX100 in the absence and presence of 4.0 × 10^−5^ M SMX and TMP at 298 K.

	SMX		TMP	
	Γ_max_ (mmol/m^2^)	A_min_ × 10^−2^(Å^2^)	Γ_max_ (mmol/m^2^)	A_min_ × 10^−2^ (Å^2^)
SDS	5.23	3.17	2.99	5.55
TX100	2.55	6.51	3.02	5.50

		No drugs		

	Γ_max_ (mmol/m^2^)		A_min_ × 10^−2^(Å^2^)	

SDS	3.66		4.54	
TX100	2.47		6.70	
