# Supplementary figures and images for: A Study on Solubilization of Poorly Soluble Drugs by Cyclodextrins and Micelles: Complexation and Binding Characteristics of Sulfamethoxazole and Trimethoprim
Source: ScientificWorldJournal. 2012 Apr 29;2012:718791. doi: 10.1100/2012/718791 (PMC3353312; doi:10.1100/2012/718791)

Figure 1. Inclusion complex formation of TMP/  $\gamma$ -CD and SMX /  $\beta$ -CD

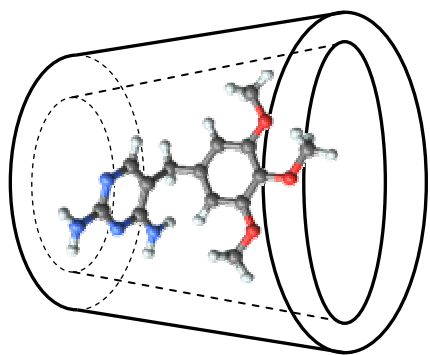

TMP/  $\gamma$ -CD

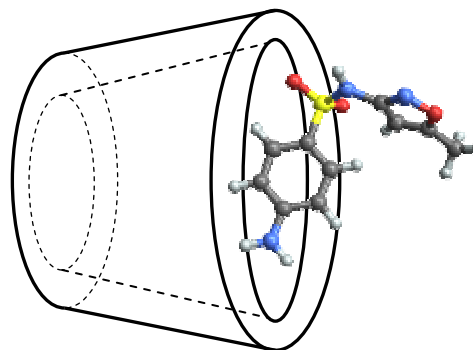

SMX /  $\beta$ -CD

Supplement: Supplementary file 1 — Inclusion complex formation of TMP/ g–CD and SMX/ b–CD (Figure 1) and micellar solubilization of TMP and SMX by SDS micelles (Figure 2). [file 718791.f1.pdf]

Figure 2. Micellar solubilization of TMP and SMX by SDS micelles

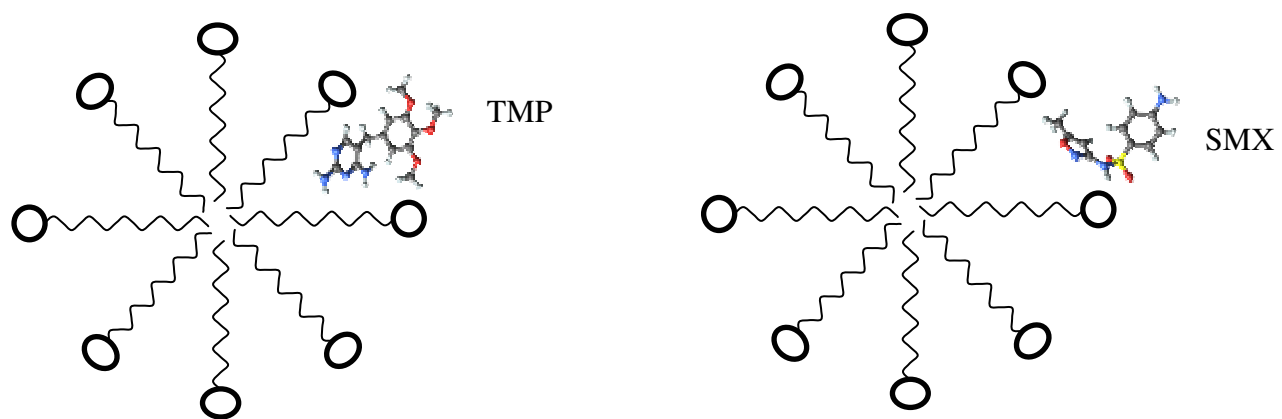

Supplement: Supplementary file 2 [file 718791.f2.pdf]
